# In-Person versus Virtual CEASE Smoking Cessation Interventions

**DOI:** 10.31586/jbls.2024.1107

**Published:** 2024-11-04

**Authors:** Payam Sheikhattari, Rifath Ara Alam Barsha, Chidubem Egboluche, Adriana Foster, Shervin Assari

**Affiliations:** 1 Prevention Sciences Research Center, Morgan State University, Baltimore, MD, USA; 2 School of Community Health & Policy, Morgan State University, Baltimore, MD, USA; 3 Johns Hopkins University, Baltimore, MD, USA; 4 Charles R Drew University of Medicine and Science, Los Angeles, CA, USA

**Keywords:** Smoking Cessation, Underserved Populations, Low-Income Smokers, Curriculum, Peer Motivation, Virtual Interventions, Self-Help, In-Person Intervention, Hybrid Intervention

## Abstract

**Background:**

Smoking cessation interventions are critical for underserved populations, particularly among low-income individuals who may benefit from tailored support. However, the effectiveness of different intervention formats remains unclear, particularly as virtual and hybrid models gain popularity.

**Aims:**

This study compares the effectiveness of three smoking cessation intervention arms in a quasi-experimental design: Self-help group (Arm 1), In-person group (Arm 2), and Virtual/hybrid group (Arm 3). The primary outcome was the rate of successful quit across these different intervention modalities.

**Methods:**

The study utilized a community-based intervention approach, controlling for potential confounders. The communities were randomized, and this process was blinded. The effectiveness of the In-person group and the Virtual/hybrid group was compared to the Self-help group. The odds ratio (OR) for successful quit rates was calculated for each group, with corresponding 95% confidence intervals (CIs).

**Results:**

Participants included 50.4% of women, 82.8% were Black Americans, 11.6% Whites, and 3.4% other races. In-person group (Arm 2) showed a higher rate of successful quit compared to the Self-help group (OR = 2.67, 95% CI = 1.05, 6.79). Virtual/hybrid group (Arm 3) was not associated with a significantly higher quit rate compared to the Self-help group (OR = 1.48, 95% CI = 0.57, 3.83).

**Conclusion:**

The In-person group, which utilizes the CEASE curriculum and incorporates peer motivation, proved to be significantly more effective than both the Self-help and Virtual/hybrid groups. The findings suggest that low-income, underserved smokers may not be fully prepared to benefit from virtual interventions, or the current curriculum may need adaptation to better serve their needs in a virtual format.

## 1. Background

Smoking is the number one preventable killer in the U.S., responsible for more than 480,000 premature deaths each year, including more than 41,000 from secondhand smoke [1,2]. As such, we need effective Smoking Cessation Interventions to reduce the burden of tobacco use in the U.S. population.

Over the past few decades, tobacco use within the general U.S. population has been steadily decreasing, from 42.4% in 1965 to 15.1% in 2015 [2]. This substantial reduction is often attributed to the development of various treatment options, including nicotine replacement therapy (NRT), which works to diminish chemical dependency by substituting nicotine from cigarettes with NRT products and gradually tapering down the dosage [3]. Additional contributing factors include the introduction of smoking cessation aids such as varenicline and bupropion, psychological counseling, the implementation of tobacco counter-marketing policies, and the enactment of clean indoor air laws [2,4,5]. However, despite these advances, health disparities related to tobacco use have continued to widen. Across the state of Maryland, it is estimated that 15.1% of adults are smokers [1]. Recent data collected in low income areas of Baltimore through CEASE (Communities Engaged and Advocating for a Smoke-free Environment) surveys indicate that smoking rates are substantially higher in certain communities, with prevalence ranging from 27% among parents of school-age children to as high as 75% among adults surveyed at a community market [6].

Therefore, there is a need for culturally sensitive cessation programs that include essential components such as education, counseling, and social support. This is because individuals who are more informed, better prepared, and are highly motivated to alter their behavior have significantly better chances of success in quitting [7]. Social support, in particular, plays a critical role in motivating and helping former smokers manage the social norms and environmental cues that previously reinforced their smoking behavior [8], ultimately facilitating positive changes. Nonetheless, reducing tobacco use within underserved communities has proven to be an ongoing challenge. In the U.S., only 4 in 10 smokers make an attempt to quit each year and since they do so without utilizing evidence-based treatments, most of them relapse within six months [7,9]. Individuals from racial/ethnic minority communities who also have low income and lower levels of education are even less likely to seek assistance from healthcare providers or to take advantage of proven interventions such as face-to-face counseling, smoking cessation clinics, medications, and quit lines [10]. Moreover, many health interventions, including clinical trials for substance abuse, often exclude populations facing health disparities due to strict eligibility criteria. While such interventions may demonstrate efficacy in carefully controlled circumstances, their success is far less certain when applied to populations that are economically disadvantaged, less educated, or socially marginalized. Therefore, while these treatments may be effective under specific conditions, there is a high likelihood that they will fail when implemented in these vulnerable populations.

At the same time, the rapid expansion and adoption of digital health tools have created new opportunities to reach patients who previously faced significant barriers to care [11]. The transition to virtual care was arguably less dramatic for tobacco use treatment services, as telephone consultations have long been a fundamental treatment method in community and clinical environments [12]. These tools augment the potential for participation and bidirectional communication between patients and providers, both asynchronously and synchronously; hence, they boost patient engagement and empowerment. Virtual care technologies are increasingly being utilized as an efficient and cost-effective method for delivering quality healthcare services to patients in rural areas or areas with provider shortages [12,13]. It enhances efficiency without incurring extra net costs, reduces patient travel and wait times, and allows comparable or improved quality of care, thereby increasing patient and provider satisfaction with care [12].

The utilization of online technologies and communication resources remains insufficient in underserved low-income communities of color. Studies have suggested that digital divide may be yet another social determinant of health [14–16]. This is primarily due to factors such as limited access to the necessary resources, lack of reliable internet connectivity, a lack of awareness of available digital tools, and lower levels of self-confidence in using these technologies [14,15]. Thus, both the reach and utilization of effective tobacco cessation treatments among less advantaged populations remain suboptimal.

Communities Engaged and Advocating for a Smoke-free Environment (CEASE), initiated in 2007, is a Community-Based Participatory Research (CBPR) partnership committed to addressing tobacco-related health disparities in underserved urban communities initiated with funding support from the National Institute on Minority Health and Health Disparities (NIMHD). During the past ten years, CEASE has surveyed more than 6,000 individuals, designed and tested four phases of community-based smoking cessation interventions, launched school- based prevention initiatives, and developed CEASE branded smoking cessation tools for peer-motivation and education [17–20]. CEASE-1 [17, 21, 22], CEASE-2 [19, 22, 23] and CEASE-3 [19,22,23] have shown efficacy of this program in different settings, using different modes of intervention. In the first phase, cessation services were provided in a health clinic; in Phases II, III, and IV services were provided in community venues [19]. The CEASE 1 to 3 all used a 12-week curricular intervention, while the CEASE-4 is an attempt to have a fewer number of sessions [24]. CEASE has been able to collaboratively involve a diverse group of partners in designing and implementing a culturally appropriate smoking cessation curriculum and intervention. Less is known about best practices for community use of virtual care technologies [16].

Built on our previous iterations of CEASE program, our specific aim is to assess the effectiveness of in-person versus virtual versions of a smoking cessation peer-motivation intervention in terms of their success rates (quitting and staying quit). For this aim, our primary hypothesis is that the smoking cessation rate will be equal to or higher in the virtual peer-motivation arm (non-inferiority trial) than the in-person and self-help/control arms.

## 2. Methods

### 2.1. Study Design and Settings

The current study employed a randomized cluster trial design. Three Baltimore City communities were randomly allocated to one of three arms of the study: 1) In-person intervention, 2) Virtual/hybrid intervention, and 3) Self-help/control group. To minimize bias, randomization was blinded and conducted during a steering committee meeting. The three communities selected for this study were Oldtown/Middle East, Waverly, and Southwest (Poppleton/The Terraces/Hollins Market/Washington Village/Pigtown) and shared similar sociodemographic profiles and resources, such as schools, faith-based organizations, and healthcare centers, which enhanced their comparability. Initially, the program launched a fully virtual arm instead of a hybrid one. However, due to logistical issues, the virtual arm was later modified to a hybrid format. In addition, we did have to expand the neighborhood boundaries to support our recruitment efforts. Those outside of those original boundaries were randomized by site.

### 2.2. Ethical Consideration

The study received approval from Morgan State University’s Institutional Review Board (IRB #19/06-0082). All participants provided informed consent prior to enrollment. To ensure confidentiality, each participant was assigned a unique identification number, which was kept separate from their contact information.

### 2.3. Study Participants

The inclusion criteria for the CEASE Digital smoking cessation program were: 1) age 21 years or older, 2) current smoker (smoke three or more cigarettes everyday), 3) willing and ready to quit using tobacco, and 4) providing consent to participate. Individuals enrolled in the fully virtual intervention were required to have access to devices (desktop, laptop, tablet, etc.) with reliable and consistent internet or cellular data to be eligible. This criterion was not applied if the classes were conducted in hybrid mode. Individuals with health conditions preventing them from providing consent were excluded from the study.

### 2.4. Intervention

Nine peer motivators were selected and trained to facilitate the smoking cessation classes. Some peer motivators were former smokers who had quit at least 12 months earlier and remained smoke-free. Others has some personal connection tobacco use, such as having a family history of smoking or witnessing a loved one navigate a tobacco-related illness. All the peer motivators attended a three-day workshop that covered training on CEASE Digital tobacco cessation curriculum, integration of digital platform and curriculum, human subject research ethics, study procedures and data management, and group facilitation. They were actively involved in the recruitment and enrollment of participants. Community venues for the smoking cessation classes and site coordinators for each facility were identified, and agreements were established with those facilities. The site coordinators served as the point of contact. Participants were recruited through community outreach, word-of-mouth, referrals, flyers, social media announcements, and community surveys. Smoking cessation classes were conducted in community settings such as community organizations, public housing sites or senior residences, churches, and other local venues. The recruitment of the participants started April 2022 and ended in September 2023.

The CEASE Digital smoking cessation curriculum was developed with input from community stakeholders and experienced peer motivators. The smoking cessation program was a seven-week initiative for in-person and virtual/hybrid groups. The in-person intervention utilized the CEASE Today Tobacco Cessation Manual, developed as part of earlier CEASE initiatives. They also received printed copies of other cessation materials and resources. For the virtual/hybrid intervention, a newly developed website with smoking cessation modules was used that mirrored the lessons of the CEASE Today Tobacco Cessation Manual. In this arm, participants were given access to the CEASE tobacco cessation website and provided with virtual materials, information, and resources by their assigned peer motivators. Fully virtual smoking cessation sessions were offered using the online platform Zoom. For hybrid sessions, the only difference was that classes were held in person rather than on Zoom. Each session lasted for 2 hours. Two peer-motivators were assigned for each cohort.

Sessions during the first two weeks were held in person for both the in-person and virtual/hybrid groups. In the first week, participants went through the informed consent process facilitated by peer motivators in a group setting. Orientation packets containing relevant materials were distributed to participants. In the second week, class information and technology training (using Zoom) were provided. The smoking cessation classes using the curriculum commenced in week three and continued with a total of five sessions delivered weekly over five weeks. Weeks three and four focused on motivating and preparing participants to quit smoking, weeks five and six were dedicated to quitting, and week seven focused on preventing relapse.

Participants in the self-help/control group received the services already in place, including a one-hour motivational enhancement and educational session, self-help smoking cessation materials (a hard copy of the CEASE Today Tobacco Cessation Manual and a link to the CEASE web materials), and other available resources, including information about local tobacco cessation services.

### 2.5. Questionnaires

We developed a baseline questionnaire that captured participant information on socio-demographics, smoking history and status, physical, behavioral, and mental health, perceived social support, and other variables. All participants in the in-person and virtual/hybrid groups were followed approximately three months after completing their smoking cessation classes to ascertain their smoking status, and they completed a follow-up survey. The self-help/control group participants completed the follow-up survey five months after their enrollment.

### 2.6. Measures

#### 2.6.1. Outcome Variable

The outcome of the study was smoking status at follow-up. The variable was used as a binary measure (0=Did not quit, 1=Quit).

#### 2.6.2. Predictors

The study included several predictors: age, race, gender, intervention arm, and Fagerstrom score. Age was categorized as 50 years or less versus more than 50 years, while gender was treated as a binary variable (0=male and 1=female). Self-reported race was classified into three categories: Black, White, and Other/Multiple, with the latter encompassing American Indian/Alaska Native, Asian, and individuals identifying with multiple races. The Fagerstrom Nicotine Dependence Test, a standard instrument used to evaluate physical nicotine dependence [25,26], was utilized to measure participants’ nicotine dependence at baseline. The score ranges from 0 to 10, and a higher score indicates higher addiction. The intervention arm was used as a categorical variable (1=In-person, 2=Virtual/hybrid, and 3=Self-help/control).

Educational attainment (1 = Some high school or less, 2 = Graduated from high school/GED, 3 = Some college, 4 = Bachelor’s degree or more), family income (1 = Less than $25,000, 2 = $25,000 or more), employment status (0 = No, 1 = Yes), marital status (0 = Not married, 1 = Married), general health (0 = Poor/Fair, 1 = Good/Excellent), alcohol abuse/dependence (0 = No, 1 = Yes), drug addiction/abuse/dependence (0 = No, 1 = Yes), problems experienced during the CEASE class (0 = No, 1 = Yes), depression, perceived stress, perceived social support, CEASE class satisfaction, and helpfulness of the CEASE class were presented in the descriptive table but were not included in the regression models.

Depression was measured using the Patient Health Questionnaire 2 (PHQ-2), which is a reliable and valid measure of depression [27]. Each item was evaluated based on the frequency of the symptom over the preceding two weeks, with participants responding on the following scale: 0 = not at all, 1 = several days, 2 = more than half the days, and 3 = nearly every day. The sum score of these two items was then calculated and used as a continuous variable. The Cronbach alpha of the items was 0.88.

Perceived social support was assessed using the 8-Item Duke/UNC Functional Social Support Questionnaire (DUFSSQ)[28]. Each item was rated on a 5-point Likert scale ranging from 1 (Much less than you would like) to 5 (As much as you would like). The mean score of the eight items was calculated and used as a continuous variable. The Cronbach’s alpha for the scale was 0.91.

Perceived stress was measured using the Perceived Stress Scale-4 (PSS-4)[29]. Each item was assessed using a 5-point Likert scale, ranging from 0 (Never) to 4 (Very often). We calculated the sum of these four items, resulting in a score that ranged from 0 to 16. The Cronbach’s alpha for the items was 0.51.

CEASE class satisfaction was measured by asking the participants the following question: How Satisfied are you with the peer motivation you received, on a scale of 1-10? (1= not at all satisfied and 10 = extremely satisfied). This variable was treated as a continuous measure

Helpfulness of the CEASE class: CEASE asked the participants the following question to assess how useful the classes were based on their experience: On a scale of 1-10 (1= not at all useful and 10 = extremely useful), how useful were the CEASE classes? This variable was treated as a continuous measure.

### 2.7. Statistical Analysis

We conducted univariate analysis to review each variable (means and proportions). Bivariate analysis was performed to examine predictors by study arm. The Chi-square test was used for categorical variables, and the Kruskal-Wallis test was used for continuous variables. Unadjusted and adjusted logistic regressions were conducted to examine the relationship between several predictors (age, gender, race, study arm, and Fagerstrom score) and quit status. The logistic regression results were presented as odds ratios (ORs), 95% confidence intervals (CIs), and significant p-values. The significance level was set at p < 0.05. We used Stata 15.0 (StataCorp LLP) for the analysis.

## 3. Results

### 3.1. Descriptive Results

This study presents data on 232 participants who completed the follow-up out of 390 participants who were recruited to the three-arm intervention. The participants’ characteristics are presented in Table 1. The majority of the participants were 50 years of age or older (78.4%). Participants included 50.4% of women, 82.8% were Black Americans, 11.6% Whites, and 3.4% other races. About 27.6% of the participants did not graduate from high school, about 60.3% had income less than $25,000, and 88.4% were unemployed.

The quit rate was highest among in-person group participants. About 26.6% of the participants enrolled in the in-person group reported quitting smoking, while the quit rate was 17.8% for the virtual/hybrid group and 14.3% for the self-help group. The participants in the three arms were not significantly different in education, employment, family income, marital status, Fagerstrom scores at baseline, stress, and social support. However, significantly more men were enrolled in the self-help arm (66.7%). The enrollment of White participants was also higher in the self-help group (23.8%). The mean depression score was lowest among the virtual/hybrid group, which was 1.0 (SD=1.5). CEASE class satisfaction was highest among the in-person group, with a mean score of 9.5 (SD=1.0). The participants in the in-person group also had the highest mean score of rating of the CEASE class helpfulness: mean=9.0 (SD=1.4).

### 3.2. Logistic Regression Results

Table 2 presents the results of the multivariable logistic regression analyses. In-person intervention showed significantly higher odds of quit (AOR=2.67, p < 0.05). Age, gender, race, and Fagerstrom score at enrollment did not show any significant association with quit smoking.

## 4. Discussion

The findings of this study provide important insights into the effectiveness of different formats of smoking cessation interventions, particularly for underserved populations. The comparison between in-person, virtual/hybrid, and self-help groups in the CEASE program highlights the superiority of in-person interventions in achieving higher quit rates among participants.

Our results clearly indicate that the in-person smoking cessation group was the most successful in helping participants quit smoking, with a 26.6% quit rate, significantly higher than both the virtual/hybrid and self-help groups. These findings are consistent with prior research that emphasizes the importance of face-to-face interactions in behavioral interventions for underserved populations. The simplicity and structure of in-person sessions allows for more direct peer motivation, real-time engagement, and emotional support, which can enhance the accountability and commitment of participants.

While virtual and hybrid interventions have gained popularity, post pandemic, due to their flexibility and accessibility, our study suggests that this format may not be as effective for low-income, underserved populations. The quit rate for the virtual/hybrid group (17.8%) was not significantly higher than the self-help group, highlighting the potential challenges these populations face in engaging with digital health interventions. Barriers such as limited access to reliable internet, lower digital literacy, and a lack of comfort with technology might explain why the virtual/hybrid approach was less effective. Additionally, virtual sessions may lack the same level of peer interaction and motivation that is available in-person, which could diminish their impact on behavior change.

This study’s focus on low-income, predominantly Black and minority populations sheds light on the unique challenges these communities face in quitting smoking. Despite the availability of interventions, quit rates remain relatively low across all formats, underscoring the persistent health disparities related to smoking. Structural barriers such as unemployment, low education, and high stress levels likely play a role in reducing the effectiveness of smoking cessation programs in these communities. In addition, the lower quit rates observed in the virtual and self-help groups suggest that these populations may benefit more from high-touch, community-based interventions that provide direct support and motivation.

The results of this study highlight the need for public health strategies that prioritize in-person interventions in underserved communities, where social support and direct engagement are critical for behavior change. While virtual interventions may offer convenience and scalability, they are not always suitable for populations facing socioeconomic barriers. Tailored programs that address the specific needs of low-income smokers, such as improving access to digital tools and enhancing peer support, coupled with new Artificial Intelligent tools and applications, could help bridge the gap between the effectiveness of virtual and in-person interventions and need to be further explored.

Moreover, our findings suggest that future smoking cessation programs should consider incorporating elements that reduce the digital divide, such as providing participants with the necessary technology or training to engage with virtual interventions specially through locally trained peers providing the training and coaching services. The relatively comparable high quit rates among the self-help group may highlight the significant role of the CEASE peer motivators in providing initial training and orientation. This likely led to a more meaningful engagement with the CEASE curriculum, indicating a cost-effective intervention for addressing tobacco use among underserved populations. Lastly, programs could also explore hybrid models that combine the convenience of virtual sessions with occasional in-person meetings to maximize engagement and support.

### 4.1. Limitations

Several limitations should be considered when interpreting the results of this study. First, the study sample consisted of participants who were already willing to quit smoking, which may have introduced selection bias. Additionally, the study was conducted in specific communities in Baltimore, limiting the generalizability of the findings to other populations. Another limitation is the potential for self-reporting bias in the smoking status data, which could affect the accuracy of the quit rates. Finally, while the study controlled for some key variables, there may be unmeasured confounders, such as participants’ mental health or previous quit attempts, that could influence the results.

## 5. Conclusion

In conclusion, this study demonstrates the effectiveness of in-person smoking cessation interventions over virtual/hybrid and self-help models in underserved, low-income populations. While virtual interventions hold promise for expanding access to care, their limitations in certain populations must be addressed to improve their effectiveness. Public health efforts should focus on expanding in-person support and addressing the broader structural barriers that hinder smoking cessation in minority and low-income communities. Future research should explore innovative ways to adapt digital interventions to better meet the needs of these populations and reduce the health disparities associated with smoking.

## Figures and Tables

**Table 1.** Bivariate Analyses Comparing Participants in the Three Intervention Arms

Variables	In-person Intervention Group (n= 79)	Virtual/Hybrid Intervention Group (n=90)	Self-help/Control Group (n=63)	Total (n=232)

	n (%)	n (%)	n (%)	n (%)

Quit Smoking				
No	58 (73.4)	74 (82.2)	54 (85.7)	186 (80.2)
Yes	21 (26.6)	16 (17.8)	9 (14.3)	46 (19.8)
Age (years)*				
50 years or less	20 (25.3)	10 (11.1)	17 (27.0)	47 (20.3)
More than 50 years	59 (74.7)	79 (87.8)	44 (69.8)	182 (78.4)
Missing	-	1 (1.1)	2 (3.2)	3 (1.3)
Gender**				
Men	30 (38.0)	35 (38.9)	42 (66.7)	107 (46.1)
Women	45 (56.9)	53 (58.9)	19 (30.1)	117 (50.4)
Missing	4 (5.1)	2 (2.2)	2 (3.2)	8 (3.5)
Race*				
Black American	68 (86.1)	81 (90.0)	43 (68.2)	192 (82.8)
White	8 (10.1)	4 (4.5)	15 (23.8)	27 (11.6)
Other/Multiple	2 (2.5)	3 (3.3)	3 (4.8)	8 (3.4)
Missing	1 (1.3)	2 (2.2)	2 (3.2)	5 (2.2)
Educational Attainment				
Some high school or less	19 (24.05)	27 (30.00)	18 (28.57)	64 (27.6)
Graduated from high school/GED	29 (36.71)	32 (35.56)	21 (33.33)	82 (35.3)
Some college	20 (25.32)	20 (22.22)	14 (22.22)	54 (23.3)
Bachelor or more	11 (13.92)	9 (10.00)	8 (12.70)	28 (12.1)
Missing	-	2 (2.22)	2 (3.17)	4 (1.7)
Family Income				
Less than $25,000	40 (50.63)	60 (66.67)	40 (63.49)	140 (60.3)
$25,000 or more	11 (13.92)	8 (8.89)	3 (4.76)	22 (9.5)
Missing	28 (35.44)	22 (24.44)	20 (31.75)	70 (30.)
Employment				
No	68 (86.08)	83 (92.22)	54 (85.71)	205 (88.4)
Yes	10 (12.66)	4 (4.44)	6 (9.52)	20 (8.6)
Missing	1 (1.27)	3 (3.33)	3 (4.76)	7 (3.0)
Marital Status				
Not married	71 (89.87)	80 (88.89)	49 (77.78)	200 (86.21)
Married	8 (10.13)	8 (8.89)	12 (19.05)	28 (12.07)
Missing	-	2 (2.22)	2 (3.17)	4 (1.72)
General Health				
Poor/fair	38 (48.1)	45 (50.0)	32 (50.8)	115 (49.6)
Excellent/good	41 (51.9)	43 (47.8)	29 (46.0)	113 (48.7)
Missing	-	2 (2.2)	2 (3.2)	4 (1.7)
Behavioral Health Problems				
Alcohol abuse/dependence (Yes)***	16 (20.3)	14 (15.6)	29 (46.0)	59 (25.4)
Drug addiction/abuse/dependence (Yes)	7 (8.9)	9 (10.0)	6 (9.5)	22 (9.5)
Problem Experienced During CEASE Class**				
No	77 (97.47)	73 (81.11)	58 (92.06)	208 (89.66)
Yes	2 (2.53)	17 (18.89)	5 (7.94)	24 (10.34)
	Mean (SD)	Mean (SD)	Mean (SD)	Mean (SD)
Fagerstrom (at baseline)	4.1 (1.9)	4.4 (2.1)	4.8 (2.0)	4.4 (2.0)
Depression*	1.4 (1.9)	1.0 (1.5)	1.6 (1.7)	1.3 (1.7)
Perceived Stress Scale	5.1 (3.1)	5.0 (3.2)	5.8 (2.9)	5.3 (3.1)
Perceived Social Support	4.1 (1.0)	4.2(0.9)	3.8 (1.1)	4.1 (1.0)
CEASE Class Satisfaction***	9.5 (1.0)	8.9 (1.6)	8.2 (2.0)	8.9 (1.7)
Helpfulness of CEASE Class**	9.0 (1.4)	8.6 (1.7)	7.9 (1.9)	8.6 (1.7)

Abbreviations: SD= Standard Deviation;

*** *P* < *0.001,*

** *P* < *0.01,*

* *P* < *0.05*.

**Table 2.** Multivariable Logistic Regression Analyses of Factors Predicting Quit Smoking

Variables	Quit Smoking	
	Unadjusted OR (95% CI)	Adjusted OR (95% CI)
Study Arm		
Self-help group	Ref.	Ref.
In-person group	2.17 (0.92, 5.16)	2.67* (1.05, 6.79)
Virtual/hybrid group	1.30 (0.53, 3.16)	1.48 (0.57, 3.83)
Age		
50 years or less	Ref.	Ref.
More than 50 years	1.29 (0.56, 2.98)	1.39 (0.57, 3.42)
Race		
White	Ref.	Ref.
Black American	1.56 (0.51, 4.77)	1.32 (0.41, 4.39)
Others	0.82 (0.08, 8.60)	0.92 (0.08, 10.69)
Gender		
Men	Ref.	Ref.
Women	0.57 (0.30, 1.11)	0.44 (0.22, 0.88)
Fagerstrom (at baseline)	0.93 (0.79, 1.09)	0.55 (0.26, 1.18)

Abbreviations: 0R= Odds Ratio; CI=Confidence Interval;

* *P* < *0.05*.
